# Atypical Hepatic Hemangioma with Fluid-Fluid Level on CT and MRI: Emphasis on Added Value of Contrast-Enhanced Ultrasound Findings

**DOI:** 10.5334/jbsr.2824

**Published:** 2022-06-09

**Authors:** Ye Rin Kim, Ji Eun Lee, Min Jung Jung

**Affiliations:** 1 Soonchunhyang University College of Medicine, Bucheon Hospital, KR

**Keywords:** hepatic hemangioma, fluid-fluid level, contrast-enhanced ultrasonography, computed tomography, magnetic resonance imaging

## Abstract

We present an atypical case of a patient with hepatic hemangiomas showing fluid-fluid levels on computed tomography (CT) and magnetic resonance imaging (MRI). None of the lesions showed contrast enhancement, mimicking complicated hepatic cysts or metastasis with hemorrhagic content. On contrast-enhanced ultrasound the lesions showed peripheral nodular enhancement with complete fill-in on late phases, suggestive of hepatic hemangioma.

**Teaching point:** Contrast-enhanced ultrasound (CE-US) may be useful in diagnosing atypical hepatic hemangioma showing fluid-fluid levels on computed tomography (CT) or magnetic resonance imaging (MRI).

## Introduction

Hemangioma is the most common benign hepatic tumor, usually diagnosed without difficulty due to typical imaging features. However, atypical features of hepatic hemangiomas including fluid-fluid level have been reported [1,2,3]. When these uncommon findings are combined with extremely slow or no contrast enhancement on computed tomography (CT) or magnetic resonance imaging (MRI), differentiation from other cystic lesions, such as complicated cysts, mucinous cystic neoplasm, or even metastases with hemorrhage or necrosis may be impossible [1,4].

We present a case of hepatic hemangiomas with fluid-fluid levels on CT and MRI that was further evaluated with contrast-enhanced ultrasound (CE-US).

## Case Report

**Figure 1:**
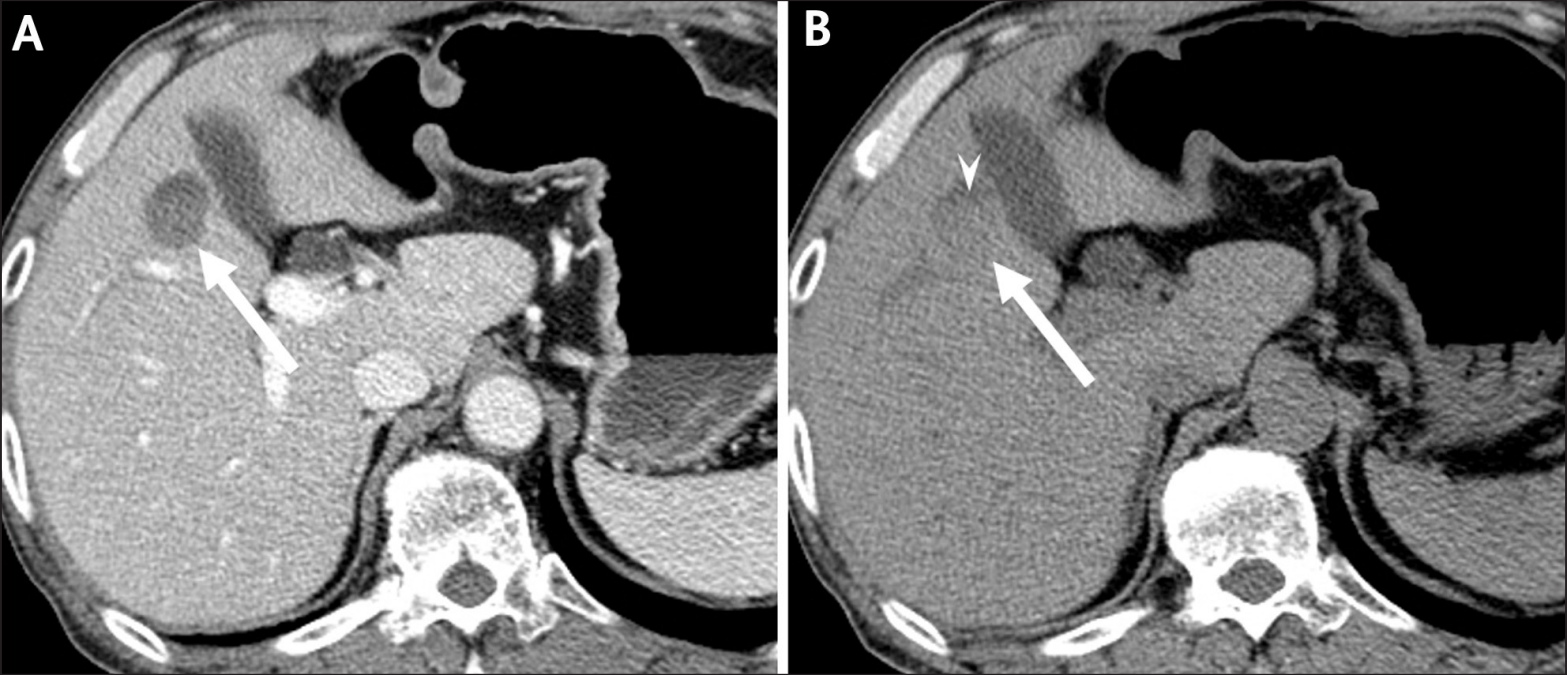
Contrast-enhanced CT image in portal phase **(A)** shows rounded, non-enhancing cystlike lesion *(arrows)* in liver segment five, with fluid-fluid level *(arrowhead)* on unenhanced CT image **(B)**.

**Figure 2:**
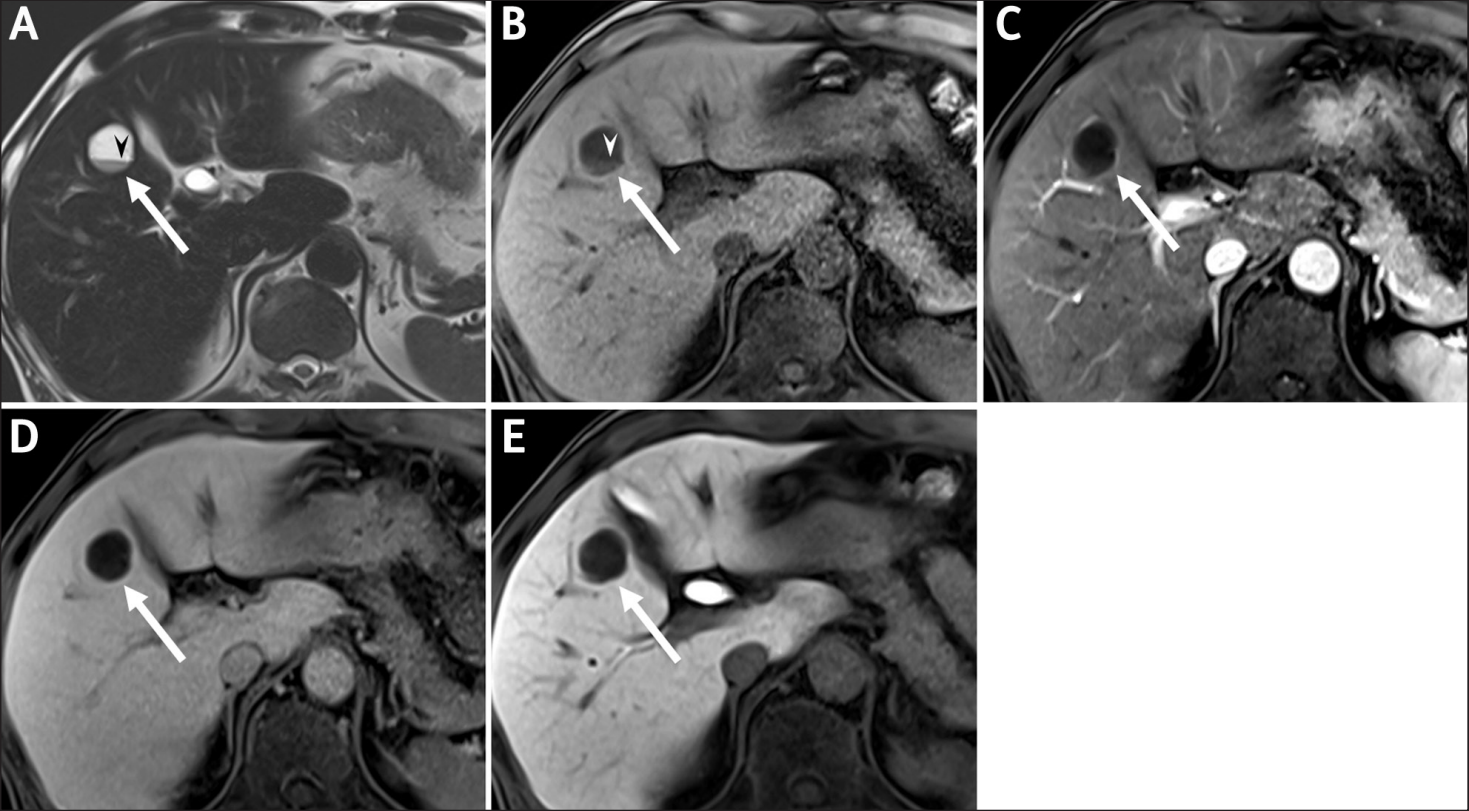
T2-weighted **(A)** and unenhanced T1-weighted MRI **(B)** show a round, well-defined cystic lesion in liver segment five *(arrows)* containing fluid-fluid level *(arrowheads)*. There is no evidence of contrast enhancement from arterial **(C)** to delayed phase **(D)**, and with signal defect on hepatobiliary phase imaging **(E)**.

A 57-year-old man was admitted for elevated liver enzymes and carbohydrate antigen 19–9. On contrast-enhanced CT, there were rounded, non-enhancing cystic lesions in liver segments four, five, and seven (Figure 1). Each lesion showed fluid-fluid levels with the lower layer of slightly higher density relative to the upper layer. On MRI, all lesions appeared cystic, showing T2 bright and T1 low signal intensity (SI), and with a fluid-fluid level. The lower layer showed T2 intermediate low and T1 subtle high SI relative to the upper layer. On gadoxetic acid-enhanced MRI, none of the lesions showed contrast enhancement, and there were signal defects on hepatobiliary phase imaging (Figure 2). The possibility of hemorrhagic cysts seemed to be most likely. However, unusual metastasis with hemorrhagic necrosis or cystic changes could not be excluded.

**Figure 3:**
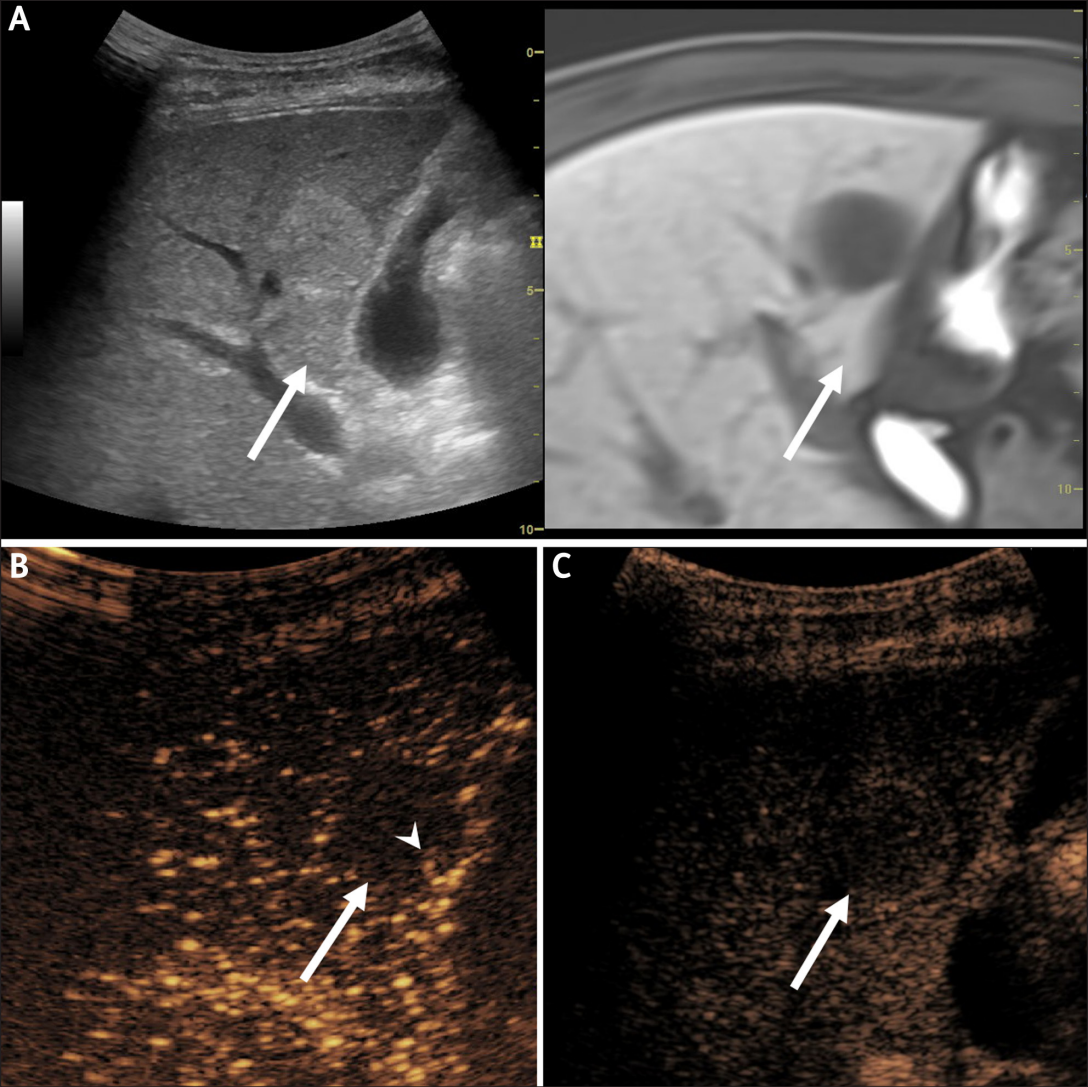
**(A)** B-mode ultrasound with MR fusion guidance shows a solid homogeneous hyperechoic lesion in liver segment five *(arrows)*. On contrast-enhanced ultrasound, **(B)** portal-phase image shows peripheral nodular contrast enhancement *(arrowhead)*, and complete fill-in and without washout on delayed-phase **(C)**.

Accordingly, ultrasound-guided biopsy was planned. In contrast to CT or MRI findings, ultrasound revealed that all lesions appeared solid with homogenous hyperechogenicity. CE-US revealed very slow peripheral nodular enhancement and complete fill-in, and without washout on delayed-phase (Figure 3). These findings were suggestive of hepatic hemangioma. Nevertheless, biopsy was performed, and pathology confirmed the diagnosis of cavernous hemangioma.

## Discussion

Fluid-fluid level may be detected when a lesion contains substances of different densities in a cystic or confined space. This feature, caused by the sedimentation effect, has been reported in several hepatic lesions, such as complicated cysts, mucinous cystic neoplasm, or metastases with cystic, hemorrhagic, or necrotic changes [1]. Fluid-fluid levels in hepatic hemangiomas have rarely been reported [1,2,3,5], yet are usually not considered in the differential diagnosis with a hepatic lesion containing fluid-fluid level.

Several reports [1,2,3] proposed that hepatic hemangiomas with extremely slow or stagnant blood flow may show fluid-fluid level with sedimentation of red blood cells in the inferior layer and serum in the superior layer. On CT, the upper layer shows fluid attenuation due to serum, while the lower shows slightly higher attenuation due to packed red blood cells. On MRI, the top layer shows fluid SI of T2 bright and T1 low SI, while the lower layer shows relatively lower T2 and slightly higher T1 SI, due to cellular content. In previous reports [2,3] hepatic hemangiomas with fluid-fluid levels showed typical enhancement pattern of a hemangioma, facilitating the diagnosis. However, in our case, there was no evidence of any contrast enhancement on CT or MRI, thus complicating the final diagnosis.

Ghai et al. [2] suggested that multiple interfaces resulting from vascular channels of the hemangioma increase the background echogenicity on ultrasound and interfere with the visualization of a fluid-fluid level. This may explain the discrepancy between the demonstration of a fluid-fluid level between ultrasound and other modalities. On CE-US, hepatic hemangioma shows the same typical enhancement pattern as on CT or MRI. About 40–50% of hepatic hemangiomas show complete fill-in during the late phase, which is considered diagnostic regardless of the B-mode appearance [6]. CE-US has advantages for its real-time operation and ability to observe the enhancement process in continuous time frame.

## Conclusion

CE-US may contribute to the final diagnosis of an atypical hepatic hemangioma with fluid-fluid levels when the characteristic hemangioma enhancement pattern is not observed on CT or MRI, and further lesion characterization is relevant for further patient management.
